# A Displacement Sensor Based on a Normal Mode Helical Antenna

**DOI:** 10.3390/s19173767

**Published:** 2019-08-30

**Authors:** Songtao Xue, Zhuoran Yi, Liyu Xie, Guochun Wan, Tao Ding

**Affiliations:** 1 Department of Disaster Mitigation for Structures, Tongji University, Shanghai 200092, China; 2 Department of Architecture, Tohoku Institute of Technology, Sendai 982-8577, Japan; 3 Department of Electronic Science and Technology, Tongji University, Shanghai 200092, China; 4 Institute of Precision Optical Engineering, School of Physics Science and Engineering, Tongji University, Shanghai 200092, China

**Keywords:** displacement sensor, helical antenna, resonant frequency, perturbation theory, normal mode

## Abstract

This paper presents a passive displacement sensor based on a normal mode helical antenna. The sensor consists of an external helical antenna and an inserting dielectric rod. First, the perturbation theory is adopted to demonstrate that both the electric intensity and magnetic intensity have a noticeable gradient change within the in-and-out entrance of the helical antenna, which will cause the sensor to experience a resonant frequency shift. This phenomenon was further verified by numerical simulation using the Ansoft high frequency structure simulator (HFSS), and results show the linear correlation between the retrieved resonant frequency and the displacement. Two sets of proposed sensors were fabricated. The experiments validated that the resonant frequency shifts are linearly proportional to the applied displacement, and the sensing range can be adjusted to accommodate the user’s needs.

## 1. Introduction

A civil structure is usually designed to fulfill its functionality for at least 50 years under service loadings and occasional situations, such as earthquakes, typhoons, explosions, and so on [1]. During its life span, both service loadings and occasional unexpected loadings may cause structural damage, such as cracks or fatigue and corrosion problems, which can lead to a catastrophic event after years of service. To guard against structural failure during long-term service, structural health monitoring has developed rapidly and has been applied widely over the last few decades [2,3,4].

Among the measurands related to a building’s structural health state, its deformation in terms of strain or displacement is a significant indicator of local damage or a failed bearing capacity of individual members. Displacement sensors are either wired or wireless. The wired sensors use cables to supply energy power and transmit data to devices such as laser transducers [5] and linear variable displacement transducers (LVDTs) [6], which usually have reliable performance, good environmental resistance, and high resolution. However, the installation of wired sensors is both time- and labor-consuming due to the cable deployment. Furthermore, the wired sensing system is prone to be undermined by human activities or natural calamities.

To facilitate the deployment of sensors, various wireless sensors are developed to discard cables for data transmission using WiFi [7], the general packet radio service (GPRS), Zigbee [8,9] or other telecommunication technologies [10]. They require an analog-digital converter (ADC) for signal conversion, a microprocessor unit (MPU) for data processing, and a wireless data transceiver for data transmission [11]. However, wireless sensors still need energy to function, either with on-board batteries or energy scavenging technologies, such as solar panels or vibrometers [12,13]. These energy supply solutions will compromise the reliability of the sensors during long-term service and increase both the complexity and the cost of sensors [11,14].

Fortunately, the power lines can be eliminated by introducing passive sensing technologies, such as the surface acoustic wave (SAW)-based [15], inductive coupled [16], and radio-frequency identification (RFID) enabled sensors [17,18,19], as well as chipless passive wireless antenna sensors [20,21]. Because these passive sensors do not have a radio on board, they consume little power harvested from interrogation waves or need no power at all. Among all wireless sensors, patch-antenna-based wireless sensors, a dielectric substrate sandwiched between a radiation patch and a ground plane, have been widely adopted in structural health monitoring [14,22,23] due to their simple configuration, multimodality, low cost and other advantages. The antenna radiation parameters, such as resonant frequency, are sensitive to temperature [24], strain [11,25,26], displacement [14,27], and cracking [17,26,28], etc. The relationship between the antenna radiation parameters and the physical measurements can be utilized as the sensing mechanism.

Many passive antenna sensors for deformation monitoring have been developed. Lopato et al. [29] designed a strain sensor based on a circular patch antenna. Both the value and direction of the strain could be measured by analyzing two different current distributions between the first and second resonant frequency. Xue et al. [30] presented a novel crack-measuring sensor based on a rectangular patch antenna fed by a pair of microstrip lines, which formed a parallel plate capacitor as a crack-sensing unit. Patch antennas with low-profile, planar substrates can be easily deployed on the surface of relevant structures, but these antennas can also pose difficulties due to the flexible substrates when they are embedded in structures.

The helical antenna provides an alternative approach for sensing displacement passively, and it has potential merit as an embedded sensor in structures. With the shape of a spiral, the helical antenna is widely used in signal transmissions [31,32,33] and circuit building [33,34] for sensing physical quantities [34,35,36].

Simons et al. [33] proposed a miniaturized inductor/antenna system for the non-contact powering of an oscillator circuit. The inductor coil is equivalent to a helical antenna and is used to transmit signals by losing power through RF radiation from the inductor. Akira et al. [34] introduced an L-C circuit formed by a capacitance and inductance (helical antenna) for retrieving wirelessly the damage index of structures. However, besides selecting inductance as the sensing part and as the information transfer part, an extra capacitance or inductance is needed to form an L-C circuit [31,32,34,37]. These sensors are large in size and trouble-prone due to the complex circuit design. To simplify the L-C circuit structure, the planar inductor-capacitor (LC) circuit has been investigated [38,39]. However, the planar inductor-capacitor circuits will have the same difficulties as patch antennas when they are embedded in structures.

In recent years, miniaturized embedded helical antennas have been proposed and applied in human body health monitoring [35,36,37]. Huang et al. [35] proposed a helical-antenna-based liquid level sensor having an internal cavity filled with liquid, whose resonant frequency is sensitive to the variation of the inside material. Murphy et al. [36] used a pseudo-normal-mode helical antenna as part of a deeply implanted wireless sensor to transmit signals. After parameter optimization, results proved that this helical antenna is an excellent candidate for being implanted. However, because these helical antenna sensors in body health monitoring often favor qualitative measurement over quantitative measurement [35], they can not meet the accuracy requirement of deformation sensing in structure health monitoring.

This paper proposes a displacement sensor based on a normal mode helical antenna. This sensor consists of an external helical antenna and an internal silicon rod. When the rod is passing through the electromagnetic field inside the antenna near the in-and-out entrance, the resonant frequencies of the sensor system will vary noticeably. Because of the compact design and encapsulated structure, it can be embedded inside concrete materials. Since the major part of the sensor is a silicon rod and a helical antenna, the cost of this antenna sensor will be extremely low.

This paper is organized as follows. Section 2 introduces the concept of the displacement sensor based on a helical antenna and illustrates the sensing mechanism using the perturbation theory. This section describes one prototype of the displacement sensor based on a normal mode helical antenna. Section 3 introduces simplified principles to create a preliminary helical antenna design. In Section 4, the appropriate dimension parameters are determined for the helical antenna and interior silicon rod. Section 5 describes the fabrication of sensors and the instrumentation setup of experiments. Conclusions are then drawn and future research potential is discussed.

## 2. Displacement Sensor Using a Helical Antenna

**Figure 1:**
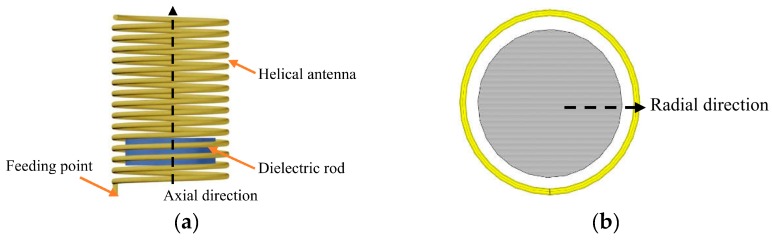
Concept of a displacement sensor using a helical antenna (**a**) Front view; (**b**) Top view.

A finite length helical antenna fed by wide band electromagnetic waves, as depicted in Figure 1, has a flat gradient of the electric intensity and magnetic intensity fields in its central part, while maintaining a steep gradient of intensities near both the in-and-out entrances of the spiral [40]. The electromagnetic field can be altered by the property variation of the objects inside the antenna [35,41], by object dislocation and material changing, while the altered field leads to the change of resonant frequency. Based on this principle, the authors propose a system consisting of a helical antenna and an inserted dielectric rod as illustrated in Figure 1, where both the helical antenna and dielectric rod are sharing the same central axial line. The electrical parameters of the system, such as the resonant frequencies, electric echo loss and current direction, vary when the dielectric rod is moving along the axis. The shift of its resonant frequencies can be selected as the distinguished feature representing the location of the rod. Then, a helical antenna displacement sensor is proposed.

### 2.1. Computational Electromagnetics Model of Proposed Displacement Sensor

The proposed helical antenna for displacement sensing works in normal mode. Under this working mode, the electromagnetic field radiated by the antenna is maximum in the radial direction and minimum along the axial direction. In order to simulate the electromagnetic property of the helical antenna, the method of moments (MOM) for computational electromagnetics can be exploited. The resulting electromagnetic field of the helical antenna can be determined by the finite element method, and so can the resonant frequencies of the helical antenna. For the sake of simplicity, the helical antenna can be modeled approximately by several small loops and short dipoles connected in a series, and the electromagnetic fields can be obtained by a superposition of the individual electromagnetic fields of each elemental loop and dipole [42]. However, several difficulties will occur while attempting to solve the Maxwell equations, which involve definition of boundary conditions and complex calculations. This makes the computational electromagnetics model inappropriate for the antenna simulation.

### 2.2. Approximation Using Perturbation Theory

Due to the similar current distribution pattern and mode of resonance, the helical antenna can be regarded as a cavity resonator for the proposed displacement sensor. In practical applications, a slight dislocation of the inside material can influence the electromagnetic field of the cavity resonator, which will consequently cause the resonant frequencies to shift. This disturbance of the cavity resonator is due to a slight change that can be approximated by the perturbation theory [41].

**Figure 2:**
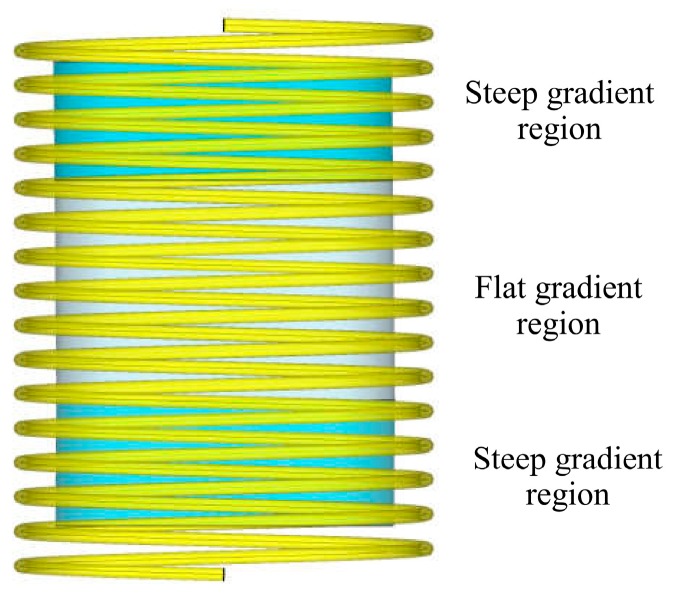
Steep and flat gradient regions of a helical antenna.

Near the in-and-out entrance of the antenna, the electric field intensity and the magnetic field intensity inside the helical antenna have a noticeable gradient change in the axial direction. We refer this area as the steep gradient region. Inside the middle area of the helical antenna, the intensities of the electromagnetic field have trivial differences along the axial direction, and this is the flat gradient region of the antenna. Finally, the electromagnetic field inside the helical antenna is divided into two steep gradient regions and one flat gradient region, which is shown in Figure 2.

**Figure 3:**
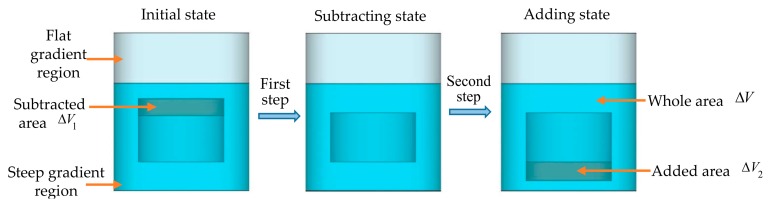
Perturbation theory flow chart of the dielectric rod in its initial state (**left**), subtracting state (**middle**), and adding state (**right**) in the steep gradient region.

The dielectric rod is moving along the axial direction, and there are two circumstances moving in the steep and flat gradient regions. Using the perturbation theory, the electromagnetic field of the helical antenna with an inserting rod from the initial position to its destination can be simulated in two steps, as illustrated in Figure 3. In the first step, the intersection volume of the inserting rod between the initial state and final state remains, while a subtracted volume is removed from the top end of the dielectric rod. In the second step, the subtracted volume is added back to the bottom end of the dielectric rod; then, the integrated volume represents the moving rod in its final position. Perturbation theory is applied to each step to simulate the electromagnetic field of the helical antenna before finally retrieving the resonant frequencies shift.

#### 2.2.1. Moving Rod in the Steep Gradient Region

In this circumstance, the dielectric rod remains entirely in the steep gradient region during the movement, which is shown in Figure 3.

In the first step, the Maxwell’s curl equations can be written for the initial state and subtracting state of the individual helical antenna as expressed here and in [41]:

(1) $$\nabla \times {\bar{E}}_{0}=-jf_{0}\mu {\bar{H}}_{0},$$

(2) $$\nabla \times {\bar{H}}_{0}=jf_{0}\varepsilon {\bar{E}}_{0},$$

(3) $$\nabla \times \bar{E}=-jf_{1}(\mu +\Delta \mu )\bar{H},$$

(4) $$\nabla \times \bar{H}=jf_{1}(\varepsilon +\Delta \varepsilon )\bar{E},$$

where ${\bar{E}}_{0}$ and ${\bar{H}}_{0}$ are the electric field intensity and the magnetic field intensity of the initial state, respectively. $\bar{E}$ and $\bar{H}$ are the fields of the subtracting state. $f_{0}$ represents the resonant frequencies of the initial state and $f_{1}$ represents the resonant frequencies of the subtracting state. j is the complex vector. μ and ε are the magnetic permeability and dielectric constant of the material, respectively. Δμ and Δε are the change of permeability and dielectric constant of the material in the subtracting area, respectively.

Multiplying the conjugate of Equation (1) by $\bar{H}$, and multiplying Equation (4) by ${\bar{E}}_{0}$; then, after subtracting these two equations, the equation can be written as

(5) $$\nabla \times ({\bar{E}}_{0}\times \bar{H})=jf_{0}\mu \bar{H}\cdot {\bar{H}}_{0}-jf_{1}(\varepsilon +\Delta \varepsilon ){\bar{E}}_{0}\cdot \bar{E}.$$

Similarly, Equation (6) can be obtained by using Equations (2) and (3)

(6) $$\nabla \times (\bar{E}\times {\bar{H}}_{0})=-jf_{1}(\mu +\Delta \mu ){\bar{H}}_{0}\cdot \bar{H}+jf_{0}\varepsilon {\bar{E}}_{0}\cdot \bar{E}.$$

Adding Equations (5) and (6), we obtain the equation by using the divergence theorem after integrating both sides over the volume $V_{0}$

(7) $$j\int_{V_{0}}\left\{\left[f_{0}\varepsilon -\omega (\varepsilon +\Delta \varepsilon )\right]E_{0}\cdot \bar{E}+\left[f_{0}\mu -\omega (\mu +\Delta \mu )\right]{\bar{H}}_{0}\cdot \bar{H}\right\}dv=0,$$

where $V_{0}$ refers to the whole volume of the cavity.

Rewriting Equation (7) and the resonant frequencies of the system will satisfy

(8) $$\frac{f_{1}-f_{0}}{f_{0}}=\frac{-\int_{V_{0}}(\Delta \varepsilon {\left|{\bar{E}}_{0}\right|}^{2}+\Delta \mu {\left|{\bar{H}}_{0}\right|}^{2})dv}{\int_{V_{0}}(\varepsilon {\left|{\bar{E}}_{0}\right|}^{2}+\mu {\left|{\bar{H}}_{0}\right|}^{2})dv}.$$

As the change of permeability and dielectric constant only exist in the subtracted area, the integral Equation (8) can be rewritten as

(9) $$\frac{f_{1}-f_{0}}{f_{0}}=\frac{-\int_{\Delta V_{1}}(\Delta \varepsilon {\left|{\bar{E}}_{0}\right|}^{2}+\Delta \mu {\left|{\bar{H}}_{0}\right|}^{2})dv}{\int_{V_{0}}(\varepsilon {\left|{\bar{E}}_{0}\right|}^{2}+\mu {\left|{\bar{H}}_{0}\right|}^{2})dv},$$

where $\Delta V_{1}$ refers to the subtracted area.

Analogously, in the second step, the resonant frequencies of the proposed sensor will satisfy

(10) $$\frac{f_{2}-f_{1}}{f_{0}}=\frac{\int_{\Delta V_{2}}(\Delta \varepsilon {\left|{\bar{E}}_{0}\right|}^{2}+\Delta \mu {\left|{\bar{H}}_{0}\right|}^{2})dv}{\int_{V_{0}}(\varepsilon {\left|{\bar{E}}_{0}\right|}^{2}+\mu {\left|{\bar{H}}_{0}\right|}^{2})dv},$$

where $\Delta V_{2}$ refers to the added area, and $f_{2}$ is the resonant frequencies of the adding state.

Based on Equations (9) and (10), we can describe the shift of the resonant frequencies of the helical antenna by the following equation

(11) $$\frac{f_{2}-f_{0}}{f_{0}}=\frac{\int_{\Delta V_{2}}(\Delta \varepsilon {\left|{\bar{E}}_{0}\right|}^{2}+\Delta \mu {\left|{\bar{H}}_{0}\right|}^{2})dv-\int_{\Delta V_{1}}(\Delta \varepsilon {\left|{\bar{E}}_{0}\right|}^{2}+\Delta \mu {\left|{\bar{H}}_{0}\right|}^{2})dv}{\int_{\Delta V_{0}}(\varepsilon {\left|{\bar{E}}_{0}\right|}^{2}+\mu {\left|{\bar{H}}_{0}\right|}^{2})dv}$$

Then the shift ratio of the resonant frequencies will satisfy

(12) $$\frac{f_{2}-f_{0}}{f_{0}}=\frac{\Delta h(c_{1}-c_{2})}{c},$$

where $c_{1}$, $c_{2}$, and c can be calculated as

(13) $$c_{1}=\int_{\Delta V_{2}}(\Delta \varepsilon {\left|{\bar{E}}_{0}\right|}^{2}+\Delta \mu {\left|{\bar{H}}_{0}\right|}^{2})dv,$$

(14) $$c_{2}=\int_{\Delta V_{1}}(\Delta \varepsilon {\left|{\bar{E}}_{0}\right|}^{2}+\Delta \mu {\left|{\bar{H}}_{0}\right|}^{2})dv,$$

(15) $$c=\int_{V_{0}}(\varepsilon {\left|{\bar{E}}_{0}\right|}^{2}+\mu {\left|{\bar{H}}_{0}\right|}^{2})dv.$$

Because the electromagnetic field inside the helical antenna is varied along the axial direction in the steep gradient region of the helical antenna, $c_{1}$ is different from $c_{2}$, which means there is a shift in resonant frequencies after the dielectric rod moves. This resonant frequency change, due to the dielectric rod moving in the steep gradient region, can be related to the displacement along the axial direction. Moreover, the rod moving in the steep gradient region can be treated as the sensing unit for the displacement. This phenomenon will be elaborated and verified by the numerical simulation in Section 3.

#### 2.2.2. Moving Rod in the Flat Gradient Region

**Figure 4:**
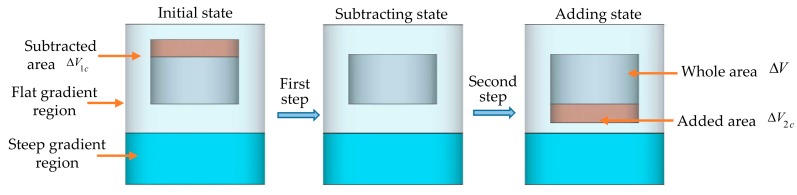
Perturbation theory flow chart of the dielectric rod in the initial state (left), subtracting state (middle) and adding state (right) in the flat gradient region.

In this circumstance, the dielectric rod remains in the flat gradient region during the movement, which is shown in Figure 4.

Analogously, the shift ratio of the resonant frequencies will satisfy Equation (12), which can be written as

(16) $$\frac{f_{2c}-f_{0}}{f_{0}}=\frac{\Delta h(d_{1}-d_{2})}{d},$$

where $f_{2c}$ is the resonant frequencies of the adding state in the flat gradient region, and $d_{1}$, $d_{2}$, and d can be calculated as

(17) $$d_{1}=\int_{\Delta V_{2c}}(\Delta \varepsilon {\left|{\bar{E}}_{0}\right|}^{2}+\Delta \mu {\left|{\bar{H}}_{0}\right|}^{2})dv,$$

(18) $$d_{2}=\int_{\Delta V_{1c}}(\Delta \varepsilon {\left|{\bar{E}}_{0}\right|}^{2}+\Delta \mu {\left|{\bar{H}}_{0}\right|}^{2})dv,$$

(19) $$d=\int_{V_{0}}(\varepsilon {\left|{\bar{E}}_{0}\right|}^{2}+\mu {\left|{\bar{H}}_{0}\right|}^{2})dv,$$

where $\Delta V_{1c}$ refers to the subtracted area and $\Delta V_{2c}$ refers to the added area.

Since the electric intensity and magnetic intensity keep steady for both subtracted and added area within the flat gradient region, $d_{1}$ and $d_{2}$ in Equations (17) and (18) are almost equivalent. That is, the shift ratio of the resonant frequencies is equal to zero while the dielectric rod is moving along the axial direction in the flat gradient region. Therefore, the flat gradient region is not appropriate for displacement sensing according to perturbation theory.

## 3. Design of the Displacement Sensor

The perturbation theory discussed in Section 2 shows that the movement of the dielectric rod would change the resonant frequencies of the helical antenna in its steep gradient region. However, how the resonant frequencies will be influenced by the movement of the dielectric rod can not be determined analytically by the perturbation theory. Furthermore, determining the dimension parameters of the sensing system will add to the computational burden of the integration in the electromagnetic field, when involving the recursive design process.

In this paper, a helical antenna will be working around 2.4 GHz for the available testing environment in the laboratory. Initially the dimension parameters of the helical antenna will be prescribed within a narrow field according to its simplified relationship with resonant frequencies. Then the computational electromagnetics method is used to finalize the dimension of the proposed sensing system.

In the design process, the chosen internal dielectric rod material is silicon to amplify the impact of the dielectric rod movement, and copper is selected as the material of the helical antenna for its excellent electrical conductivity. The basic parameters of a helical antenna are the diameter d, number of turns n, and spacing between adjacent turns h [42]. The dimension parameters of a silicon rod are length m, diameter $d_{s}$ and position of the inserting silicon rod.

### 3.1. Design of a Normal Mode Helical Antenna

For a normal mode helical antenna, the relationship between the total length of the wire and the wave length in a state of resonance can be described as [42]:

(20) $$l=\frac{(2k-1)\lambda_{p}}{4},$$

where l is the total length of the wire of the helical antenna, k is the order of resonant frequencies of the helical antenna, and $\lambda_{p}$ is the antenna’s wave length.

For the normal mode helical antenna, the resonant frequencies and the wavelengths of the antenna can be described as

(21) $$f=\frac{c}{\lambda_{p}},$$

where f is the resonant frequencies of the helical antenna.

Therefore, the total length of helical antenna wire can be approximately calculated by:

(22) l=πnd,

where d and n are the diameter and the number of turns of the helical antenna, respectively.

**Figure 5:**
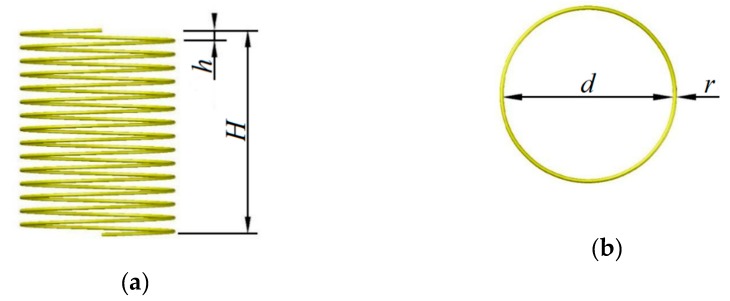
Parameters of the helical antenna (**a**) Front view; (**b**) Top view.

Based on Equations (20) and (22), we can setup the relationship between resonant frequency and parameters of the helical antenna as

(23) $$f=\frac{c(2k-1)}{4\pi nd},$$

where the diameter d and number of turns n are increasing with the order of resonant frequencies when the resonant frequencies remain constant. In this paper, the working frequency of a helical antenna is set at around 2.4 GHz. To ensure the sensing range for a helical antenna, a higher order of resonant frequency is chosen for simulation and fabrication. Based on Equation (23), the setting field for dimension parameters of the helical antenna, as explained in Figure 5, can be initially determined, and these parameters listed in Table 1. These parameters will be finalized later based on the numerical simulation.

### 3.2. Design of the Silicon Rod

**Figure 6:**
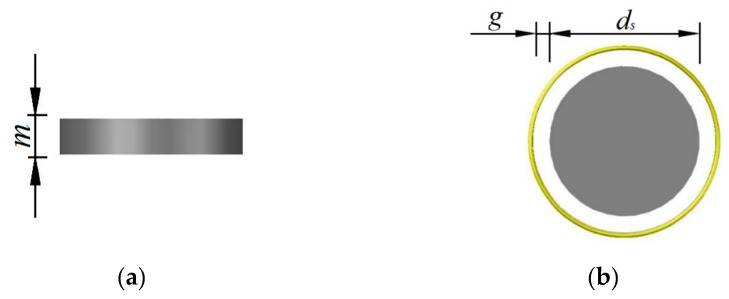
Parameters of the silicon rod (**a**) Front view; (**b**) Top view.

The basic parameters of the silicon rod are shown in Figure 6, where m and $d_{s}$ are the height and diameter of the silicon rod, and g is the distance of the gap between the silicon rod and the helical antenna in the radial direction.

The gap g is decided by the diameter of the silicon rod, which will change the volume of the added area and subtracted area. To quantify the impact of the gap, two silicon rods with different diameters, slightly less than the inner diameter of the helical antenna, are designed for comparative study. The corresponding parameters are listed in Table 2.

## 4. Modeling and Simulation

**Figure 7:**
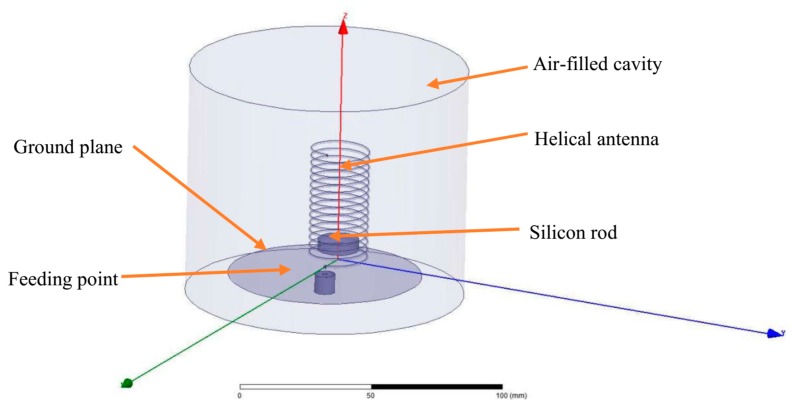
The schematic diagram of the helical antenna sensor.

The radiation properties of the helical antenna sensor are simulated using the Ansoft high frequency structure simulator (HFSS), see Supplementary Materials. The model in the HFSS consists of a helical antenna and a coaxial dielectric rod, which is shown in Figure 7. The material of the dielectric rod is silicon, while copper is chosen as the material of the helical antenna. The sensing system is arranged inside an air cylinder with a radius of about a quarter wavelength to ensure computational accuracy of the far field radiation. The helical antenna is fed by a lumped port connected with the ground plane at the end of the helical antenna. The ground plane is set as a perfect E to ensure that the electric field is perfectly perpendicular to the surface.

After calculating the 17th order resonant frequency within the setting range by HFSS, four sets of proposed sensor systems with better performances are proposed. The serial number and dimension parameters are shown in Table 3.

### Performance Simulation

**Figure 8:**
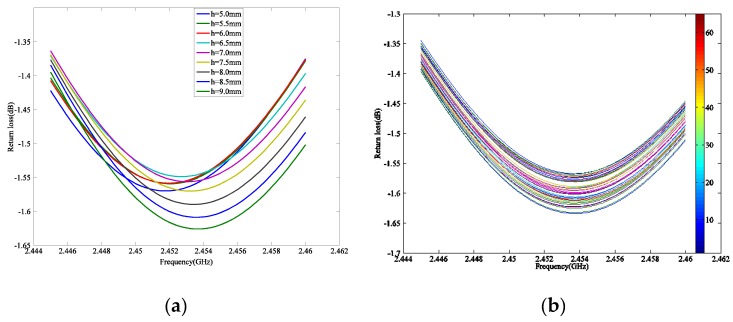
The return loss curves of TH1 group (**a**) The return loss curves from 5 mm to 9 mm; (**b**) The return loss curves from 9.5 mm to 25 mm.

The simulation proceeds in a wide range of location variations by HFSS for the TH1 group, with the location of the silicon rod moving from 5 mm above the ground plane to 27 mm. The return loss curves around the 17th order resonant frequency are acquired for each step where we insert the rod along the axis, as shown in Figure 8.

**Figure 9:**
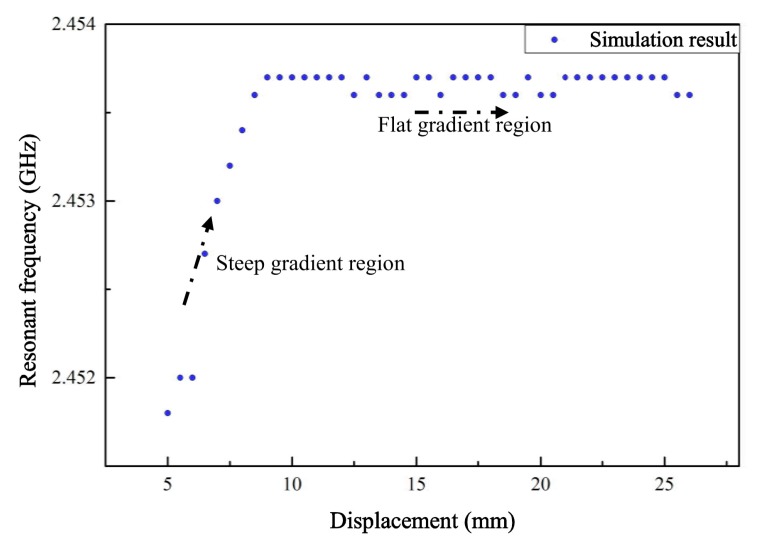
Relationship between resonant frequency and displacement based on the TH1 group in a wide range of location variations.

The 17th order resonant frequency of the displacement sensor is extracted from the return loss curve at each moving step. The scatter diagram of resonant frequencies and movement of the silicon rod (which represents the displacement of the structure) is plotted in Figure 9.

For the distance changing from 5 mm to 9.5 mm along the axial direction, the resonant frequency varies approximately linearly with the movement of the silicon rod, while the resonant frequency remains constant with the location change between 9.5 mm to 27 mm. This relationship, as shown in Figure 9, can be explained by the perturbation theory discussed in Section 2. The region between 5 mm over the ground plane to 9.5 mm inside the helical antenna can be regarded as the steep gradient region, and the region between 9.5 mm over the ground plane to 27 mm can be regarded as the flat gradient region. In this case, the height of the steep gradient region is approximately 10 percent of the total height of the helical antenna, and this area can be used for displacement sensing.

**Figure 10:**
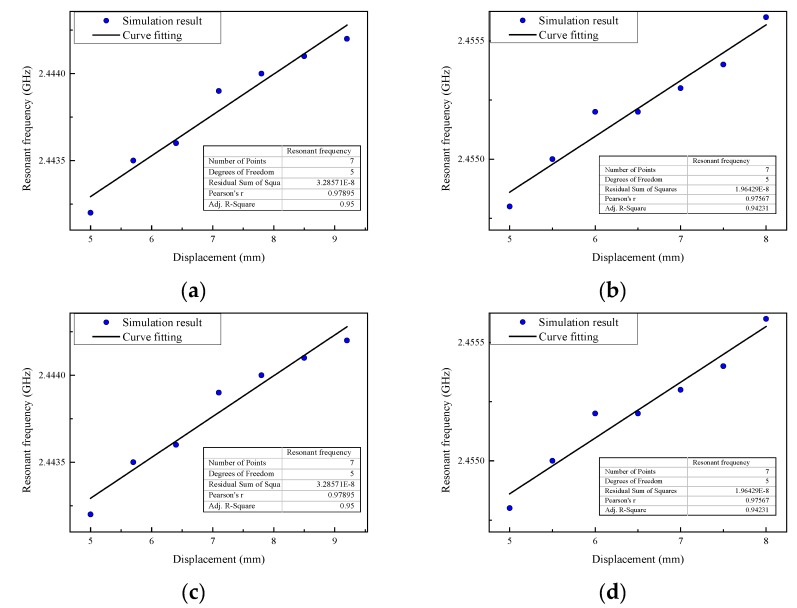
Relationship between resonant frequency and displacement for each group (**a**) Group TH1; (**b**) Group TH2; (**c**) Group TH3; (**d**) Group TH4.

Then the simulation for each group is carried out. In this stage, the silicon rod starts to move upward from the height of 5 mm until the resonant frequency does not change and the total distance is called the measuring range. The resonant frequency of the displacement sensor is extracted from the return loss curve at each movement level. The scatter diagram of the resonant frequency and movement of the silicon rod for each group are plotted in Figure 10.

Based on the simulation results, the sensitivity, measuring range and correlation coefficient of the fitted line in four test groups are shown in Table 4.

The linearity of the displacement sensor in group TH1 and group TH3 are better than group TH2 and group TH4, respectively. Moreover, there would be a better working performance with a smaller gap between the silicon rod and helical antenna. Compared with the displacement sensor in the TH3 and TH4 groups, the measuring range in the TH1 and TH2 groups decreased. The measuring range is related to the length of the steep gradient region inside the helical antenna. As the steep gradient region usually expands with the total length of the helical antenna, the measuring range would increase. In other words, we can enlarge the sensing range by increasing the height H of the helical antenna.

However, because of the differences between numerical simulation and practical conditions in radiation, surrounding environment, boundary conditions, etc., actual experiments still need to be carried out to demonstrate the performance of the displacement sensors.

## 5. Experiment

**Figure 11:**
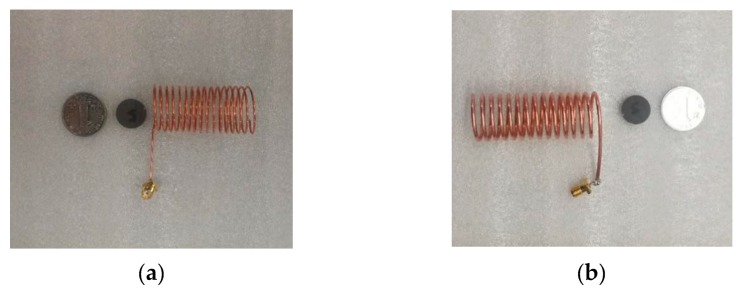
The manufactured displacement sensors (**a**) Helical antenna in group TH1; (**b**) Helical antenna in group TH3.

For the fabricated sensors, copper was selected as the material of the helical antenna and silicon with high purity was used for making the silicon rod. The two kinds of helical antennas with different silicon rods are shown in Figure 11. The parameters of the helical antenna are the same as in the simulation stage.

### 5.1. Instrumentation Setup

**Figure 12:**
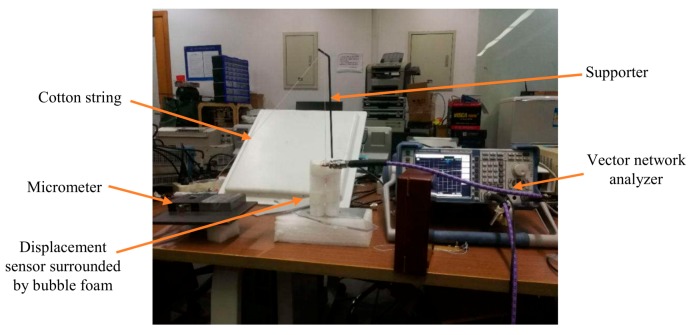
The experimental setup.

To verify the simulation results, four groups of tests were designed according to the parameters in Table 3. To ensure the coaxial movement of the silicon rod, the testing sensing system was established as shown in Figure 12. The helical antenna was surrounded by foam materials to keep it vertical. The silicon rod is suspended in the middle of the helical antenna by a cotton string. The other end of the cotton string is through the top beam; then, it connects with the micrometer, which controls the movement of the inserting rod. The helical antenna connects to the vector network analyzer (VNA- Rohde & Schwarz – Munich, Germany) via a tin-soldered Sub-Miniature-A (SMA) connector. 

The experiments were carried out for each testing group. First, the silicon rod is set 5 mm beneath the feeding point in all simulation groups. The micrometer moves horizontally with 1 mm incremental steps. As the angle between the inclined cotton string and the suspended string is roughly 45 degrees, the incremental step of the silicon rod is about 0.7 mm ignoring the angle difference in its new position. Finally, the return loss curve of the sensing system was measured five times using the vector network analyzer (VNA) after the silicon rod was stabilized for at least 3 seconds.

### 5.2. Results and Discussion

**Figure 13:**
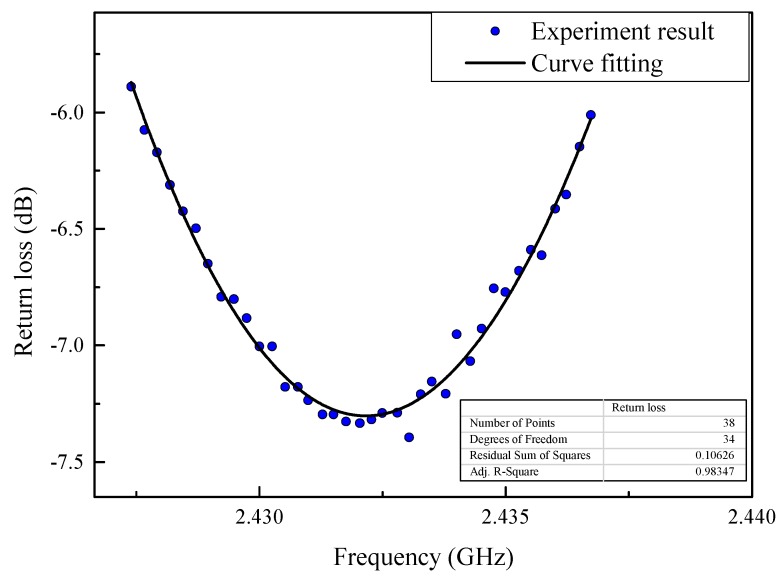
The comparison of measured points and fitted curve.

To reduce the experimental error, the return loss curves of the displacement sensor were tested five times and averaged at each incremental step. The cubic polynomial curve was utilized to fit the return loss curve around the area of desired resonant frequencies, and the resonant frequency at the local minimum was extracted for each curve. The comparison of measured points and the fitted curve around the desired resonant frequencies is shown in Figure 13.

**Figure 14:**
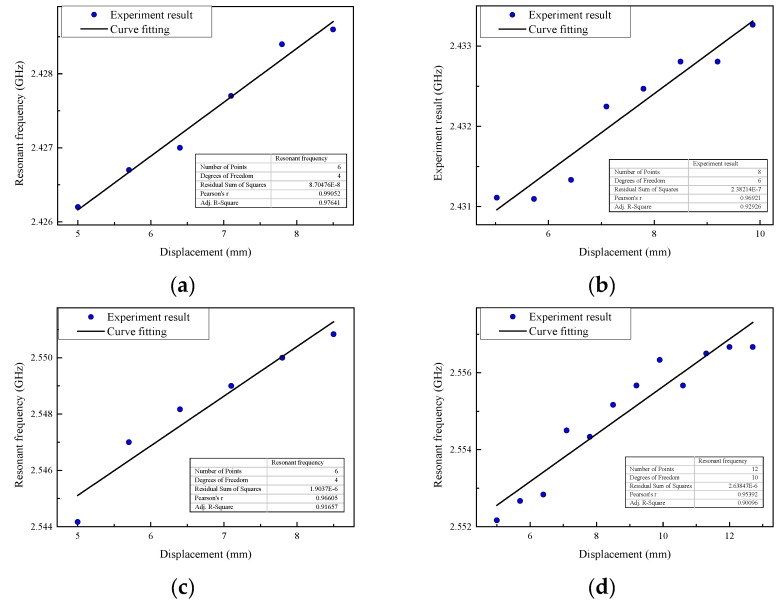
Resonant frequency with respect to displacement of the sensor in each group (**a**) Group TH1; (**b**) Group TH2; (**c**) Group TH3; (**d**) Group TH4.

Based on the experimental results, the sensitivity, measuring range and correlation coefficient of the fitted line, in four test groups, are shown in Table 5. The scatter diagram of the resonant frequency and movement of the silicon rod for each group in experiment are plotted in Figure 14.

As the correlation coefficient of the fitted line in group TH1 and group TH3 is larger than in group TH2 and group TH4, the linearity of the displacement sensor in group TH1 and group TH3 is better than group TH2 and group TH4, respectively. That is, the error caused by the gap between the silicon rod and the helical antenna will increase as the width of the gap increases, which is consistent with the simulation results.

From the experiment, it is noted that the correlation coefficient of the fitted lines of both displacement sensors is worse than that of the numerical simulation results. This difference is probably due to the following reasons.

**Figure 15:**
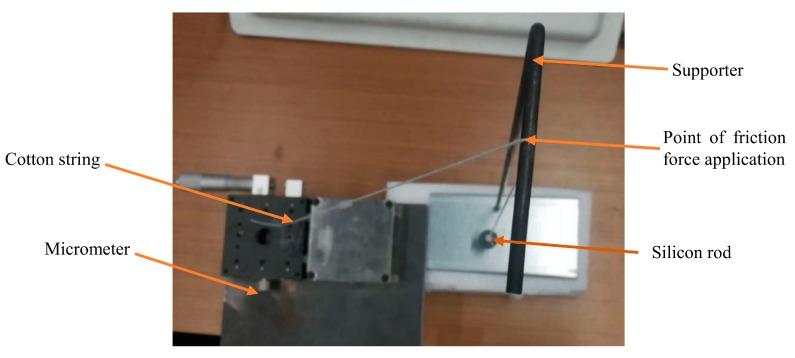
Connection between cotton string and supporter.

(1) The welding of the feeding point and environmental interference may bring in some errors that are not considered in simulation.

(2) The experiments were not controlled precisely, as the cotton string has its elasticity; furthermore, the overhead hanging point may have had side movement. The angle between the inclined cotton string and the suspended string is roughly measured, as shown in Figure 15.

(3) The silicon rod may have oscillated slightly during the measurement.

For practical use, wireless interrogation is suggested to retrieve the information for the proposed displacement sensor. A RFID chip is integrated with the helical antenna, and the resonant frequency of the sensing system can be identified by finding the active frequency with the minimum interrogation energy. Data processing methods are also needed to offset the effects caused by the interference signal and environment.

## 6. Conclusions

In this paper, a novel displacement sensing system based on a normal mode helical antenna was proposed. Using the theory of electromagnetic fields and the perturbation theory, the authors revealed a relationship between the resonant frequency of the displacement sensors and the movement of a silicon rod. Four combination sets of two types of silicon rods and two types of helical antennas were modeled and fabricated for numerical simulation and experiments. The steep gradient region inside the helical antenna proved to be useful for sensing displacement numerically and experimentally. The one-end region is about 10% of the total height of the helical antenna. The proposed helical sensor has a good performance for displacement sensing in a wired-testing environment. Results showed a sensitivity of 0.616 MHz/mm on average within a maximum effective measuring range of 7 mm, although the experimental sensitivity coefficient is susceptible to fabrication errors and environmental interference.

## Figures and Tables

**Table 1:** Setting domain for parameters of the helical antenna.

Parameters	H (mm)	h (mm)	d (mm)	r (mm)	k	n	Material
Dimensions	35–57	2–3	22.5	0.5-1	17	15	Copper wire

**Table 2:** Parameters of silicon rod (Unit: mm).

Parameters	$d_{s1}$	$g_{1}$	$m_{1}$	$d_{s2}$	$g_{2}$	$m_{2}$
Dimensions	18	2.25	4	16	3.25	4

**Table 3:** Serial number and dimension of the test group (Unit: mm).

Serial Number	H	h	d	r	n	$d_{s}$	g	m
TH1	35.5	2	22.5	0.5	15	18	2.25	4
TH2	35.5	2	22.5	0.5	15	16	3.25	4
TH3	57	3	22.5	1	15	18	2.25	4
TH4	57	3	22.5	1	15	16	3.25	4

**Table 4:** Simulation results of the test groups.

Serial Number	Sensitivity (MHz/mm)	Measuring Range (mm)	Correlation Coefficient of the Fitted Line
TH1	0.330	3.0	0.9500
TH2	0.267	3.0	0.9423
TH3	0.467	4.5	0.9665
TH4	0.500	4.0	0.9501

**Table 5:** Simulation results of the test groups.

Serial Number	Experiment			Simulation		
	Sensitivity (MHz/mm)	Measuring Range (mm)	Correlation Coefficient	Sensitivity (MHz/mm)	Measuring Range (mm)	Correlation Coefficient
TH1	0.712	3.5	0.9764	0.333	3	0.9500
TH2	0.400	4.9	0.9293	0.267	3	0.9423
TH3	1.600	3.5	0.9166	0.467	4.5	0.9665
TH4	0.650	7.7	0.9166	0.500	4	0.9501

## Supplementary Materials

The following are available online at https://www.mdpi.com/1424-8220/19/17/3767/s1.

## Abbreviations

**Symbol**	**Parameter Definition**
${\bar{E}}_{0}$	electric field intensity of the initial state
${\bar{H}}_{0}$	magnetic field intensity of the initial state
$\bar{E}$	electric field intensity of the subtracting state
$\bar{H}$	magnetic field intensity of the subtracting state
$f_{0}$	resonant frequencies of the initial state
$f_{1}$	resonant frequencies of subtracting state
μ	magnetic permeability
ε	dielectric constant
Δμ	change of magnetic permeability
Δε	change of dielectric constant
j	complex vector
$V_{0}$	volume of the cavity
$\Delta V_{1}$	subtracted area in the steep gradient region
$\Delta V_{2}$	added area in the steep gradient region
$f_{2}$	resonant frequencies of the adding state in the steep gradient region
$f_{2c}$	resonant frequencies of the adding state in the flat gradient region
$\Delta V_{1c}$	subtracted area in the flat gradient region
$\Delta V_{2c}$	added area in the flat gradient region
d	diameter of a helical antenna
n	number of turns of a helical antenna
h	spacing between adjacent turns of a helical antenna
m	length of a silicon rod
$d_{s}$	diameter of a silicon rod
l	total length of the wire of helical antenna
k	order of resonant frequencies of the helical antenna
$\lambda_{p}$	wave length of helical antenna
f	resonant frequencies of the helical antenna in design occasion
